# Health Literacy, Self-Efficacy and Glycemic Control in Patients With Diabetes Type 2 in a Greek Population

**DOI:** 10.7759/cureus.55691

**Published:** 2024-03-06

**Authors:** Panagiotis Panagiotidis, Athena Kalokairinou, Chara Tzavara, Anastasia Michailidou, Venetia-Sofia Velonaki

**Affiliations:** 1 Nursing, National and Kapodistrian University of Athens, Athens, GRC; 2 Outpatient Diabetes Clinic, General Hospital of Drama, Drama, GRC; 3 Biostatistician, School of Medicine, National and Kapodistrian University of Athens, Athens, GRC

**Keywords:** hba1c, glycemic control, type 2 diabetes mellitus, self-efficacy, health literacy

## Abstract

Aim

To investigate the relationship between health literacy (HL), self-efficacy (SE), and achievement of treatment goals in patients with type 2 diabetes mellitus (T2DM).

Method

The cross-sectional study was conducted with a random sample of patients with T2DM attending the diabetology clinic and the Home Care department of the General Hospital of Drama, Greece. They completed two questionnaires: the short form of the European Health Literacy Survey Questionnaire (HLS-EU-Q16) to measure HL and the Diabetes Management Self-Efficacy Scale (DMSES) for people with T2DM to measure SE. Medical history, demographic characteristics, and values related to glycemic control were also recorded. Linear regression analysis was used to search for the dependence of glycosylated hemoglobin (A1C) values with HL and SE and the dependence between them.

Result

About 120 patients with T2DM (response rate of 92.3%) were enrolled in the study. The mean age of the participants was 62.5 years [standard deviation (SD) = 10.6 years] and most of them were female (53.3%). A1C was found to be significantly negatively associated with diet, physical activity, and SE score. Also, a statistically significant positive correlation was found between HL and SE. HL was correlated with age, gender, education level, and A1C, with women and older people having lower HL, while conversely higher education level was significantly associated with higher HL. Higher A1C was significantly associated with lower HL. Also, SE partially mediates the relationship between HL and A1C, in a significant way.

Conclusion

The results of the study confirm the important role of HL and SE in the successful management of T2DM. Multi-level educational interventions for diabetic patients could improve HL and SE and promote diabetes self-management.

## Introduction

Diabetes mellitus is a disease with a high global prevalence. Its prevalence is constantly increasing in developed and especially in developing countries, imposing additional costs on health systems, and individual and family budgets [1]. People with diabetes are at high risk of the chronic complications of the disease and the impairments and disabilities associated with it. To prevent or delay the onset of complications, good glycemic control is required [2,3].

The determination of glycosylated hemoglobin (A1C) is the index for the assessment and monitoring of the medium and long-term (two to three months) glycemic control of the person with diabetes and is the main factor on which the treatment is based [4]. American Diabetes Association recommends an A1C goal of < 7% (53 mmol/mol) for nonpregnant adults [5]. Scientific studies have shown that achieving such therapeutic goals is directly related to proper education of patients in self-management of the disease [6-8]. It is a dynamic process of interaction between patients and healthcare professionals and requires the active participation of patients in the therapeutic process, as well as the acquisition of knowledge and skills that will allow them to self-manage the disease. Education of patients with type 2 diabetes mellitus (T2DM) is one of the key principles for integrated care and is a combination of self-management education and self-management support, a set of measures that aim to actively apply the knowledge and skills acquired in daily practice. Education includes glucose self-monitoring, meal planning, medication adherence, physical activity, foot care, and stress management [9].

The term health literacy (HL) was first coined by Simonds about five decades ago. He described the triad of health behaviors, health outcomes, and health treatment and how they are associated with successful outcomes [10]. Since then, many conceptual models as well as tools for measuring HL have been developed. According to Sørensen et al., "Health literacy is linked to literacy and entails people's knowledge, motivation and competences to access, understand, appraise, and apply health information in order to make judgments and take decisions in everyday life concerning healthcare, disease prevention, and health promotion to maintain or improve quality of life during the life course" [11].

The concept of self-efficacy (SE) is rooted in Bandura's social cognitive theory, which focuses on the interaction between behavioral, personal, and environmental factors in health and chronic disease. He described SE as the confidence that a person shows in their ability to manage a situation themself, taking and carrying all the necessary actions to reach the achievements of their goals [12,13].

The available research shows that people with chronic diseases often have low levels of HL and face a range of problems in managing health-related information [14]. Also, SE can improve outcomes and quality of life for patients living with chronic illness [15]. According to Lu et al. SE for chronic disease management partially mediated the relationships between HL and health outcomes [16]. HL and SE are concepts associated with the successful management of diabetes, too [17,18]. There is no agreement among different studies on the relationship between HL and SE in patients with diabetes as well as HL and A1C [19]. Some researchers found no association between HL and SE in patients with diabetes [20,21], while others found a significant positive association [22]. The relationship between HL and A1C is also in question because of the inconsistency of study findings. In some studies, higher HL leads to lower A1C [23] and inadequate HL was an independent predictor of poor glycemic control [24]. In others no correlation was found [25] or there was an indirect effect in A1C through its relationship with SE [22]. Review studies have shown that about one in three patients with diabetes have limited HL [26,27] and that HL is a key point in diabetes self-management [28].

To the best of our knowledge, only a few studies have examined the HL and SE in patients with T2DM and their association with the achievement of treatment goals while none of them has been conducted in Greece. Thus, this study aims to investigate the relationship of HL with SE and the achievement of treatment goals based on A1C in the Greek population.

## Materials and methods

Study design and participants

For the purpose of this cross-sectional study, 130 patients were randomly selected from among those attending regular check-ups at the outpatient diabetes clinic of the Hospital of Drama in Greece. Concerning the sample size of the study, it was calculated that with the sample size of 120 participants, the study will have 95% power to perform a linear regression analysis, at a significance level of 0.05 and for identifying effect sizes of 0.2 or greater. The outpatient diabetes clinic of the Hospital of Drama in Greece receives an average of 3000 visits from 800 patients with T2DM per year. The prefecture of Drama has approximately 95,000 inhabitants and is mainly semi-urban and rural. Every Tuesday, 15 patients come to the clinic for a regular check-up with a scheduled appointment. To ensure a random sample, five patients were selected from the list. 

Inclusion Criteria

The inclusion criteria were defined as men and women with T2DM aged over 18 years with permanent residence in the city of Drama and lowland villages of the prefecture and relative ability to understand basic instructions.

Exclusion Criteria

People with severe mental illness, mental diseases, and disabilities that impede communication were excluded from the study.

Tools

A questionnaire was used for data collection in which the demographic characteristics of the population, the comorbidity, and complications of diabetes, as well as A1C, preprandial, and postprandial blood glucose values were recorded. Height and weight measurements were taken and body mass index (BMI) was calculated.

Several tools have been developed to assess SE. One of the most widely used is the Diabetes Management Self-Efficacy Scale (DMSES), which consists of 20 questions [29]. For this study, the Greek version of the DMSES was used. It contains four factors: diet (nine items), medical therapy (five items), medication and feet check (three items), and physical activity (three items). The participant has to select from a five-point scale how convinced they are that they can perform the task described in each item. The Cronbach’s alpha coefficients were 0.93 overall and 0.92, 0.76, 0.70, and 0.79 for each factor respectively [30].

The Greek version of the short form of the European Health Literacy Survey Questionnaire (HLS-EU-Q16) was used to measure HL [31,32]. The Cronbach's alpha value has been found 0.87 [32]. The original version of the scale resulted from a review of the literature and an attempt to unify 12 conceptual models into a new comprehensive one [11]. It is focused on the cognitive aspects of HL and distinguishes four types of competencies: the ability to access, understand, appraise, and apply health information. This instrument consists of 16 questions, to be answered on a 4-point Likert scale. Participants were assigned to three HL categories: persons with adequate HL (13 or more points on the HLS-EU-Q16 scale), persons with problematic HL (9-12 points), and persons with inadequate HL (eight or fewer points).

Ethical issues

Ethical approval to conduct the research was graded by the ethics committee of the institutions involved. Patients who met the entry criteria, after being informed of the purpose of the study, gave signed informed consent to be included in the sample. Data were pseudonymized and confidentiality was ensured.

Statistical analysis

Quantitative variables were expressed as mean values, standard deviation (SD) and median (interquartile range), while qualitative variables were expressed as absolute and relative frequencies. Mann-Whitney test was used due to non-distribution for the comparison of continuous variables between two groups. Spearman correlation coefficients (rho) were used to explore the association of two continuous variables. For the mediation effect check Baron and Kenny method was used [33]. According to them, for one variable to mediate the relationship of two other variables, three conditions must be fulfilled: (1) the independent variable should be significantly associated with the dependent one; (2) the independent variable should be significantly associated with the mediator and (3) the mediator should be significantly associated with the dependent variable. Moreover, if, after the addition of the mediator in the analysis, the effect of the independent variable on the dependent one diminished, a partial mediation effect emerges. If the effect of the independent variable on the dependent becomes insignificant, the full mediation effect emerges. In both cases, the significance of the mediating effect was tested via the Sobel test. Multiple hierarchical linear regression analyses were used in order to examine the mediating role of SE in the relationship between HL and A1C. In the first step of the analysis terms for participants' characteristics and HL score were entered. In the second step, the total SE score was also included in the model. Adjusted regression coefficients (β) with standard errors (S.E) were computed from the results of the linear regression analyses. Log transformations were made in A1C, due to non-normal distribution. Also, logistic regression was used to investigate the mediating role of SE in the relationship between HL and any complication of diabetes. Odds ratios with their 95% confidence intervals were computed from the results of the logistic regression analyses All reported p values are two-tailed. Statistical significance was set at p < 0.05 and analyses were conducted using SPSS statistical software (IBM Corp. Released 2013. IBM SPSS Statistics for Windows, Version 22.0. Armonk, NY: IBM Corp).

## Results

We approached 130 people of whom 10 refused to participate in the study. The response rate was 92.30%. Data collection took approximately six months (July - December 2021). Among the 120 participants, there were 64 women (53.3%). The participants’ mean age was 62.5 years (SD = 10.6 years).

The majority of the patients (85.8%) were married or lived with their partner, were middle/high school alumni (46.7%), and had less than 400 euros monthly income (42.5%). The basic minimum net salary in Greece in 2021 was approximately 538 euros per month. Their mean BMI was 33.3 kg/m2 (SD = 6.3 kg/m2) and 63.3% of them were obese. Almost all the patients (94.2%) suffered from a chronic disease (other than diabetes) and 44.2% had at least one complication from diabetes. Mean A1C was 7.7% (SD = 1.4) and 37.8% had lower than 7% A1C. Mean glucose before a meal was 145.3 (SD = 40.2) and after a meal was 173.2 (SD = 53.4). Also, 20.8% of them were smoking. Everyone was under some kind of treatment and 34,2% were at least under one insulin a day. Their characteristics are presented in Table 1.

**Table 1:** Participants characteristics SD - Standard Deviation, BMI - Body mass index, GLP1 - Glucagon-like peptide 1, A1C- Glycosylated hemoglobin

		N (%)
Age, mean (SD)		62.5 (10.6)
Gender		
	Men	56 (46.7)
	Women	64 (53.3)
Family status		
	Married/ Living with partner	103 (85.8)
	Unmarried/ Divorced/ Widowed	17 (14.2)
Educational status		
	None	2 (1.7)
	Primary school	52 (43.3)
	Middle/ High school	56 (46.7)
	University/ MSc	10 (8.3)
Monthly income in euros		
	< 400	51 (42.5)
	401-900	48 (40)
	9001-1500	19 (15.8)
	> 1500	2 (1.7)
ΒΜΙ (Kg/m^2^), mean (SD)		33.3 (6.3)
ΒΜΙ levels		
	Normal	4 (3.3)
	Overweight	40 (33.3)
	Obese	76 (63.3)
Chronic disease		113 (94.2)
Complications of diabetes		53 (44.2)
Treatment against diabetes		
	Tablets	66 (55)
	Tablets and insulin	9 (7.5)
	Tablets and GLP1	11 (9.2)
	Tabletes, GLP1 and insulin	9 (7.5)
	GLP1	2 (1.7)
	Insulin and GLP1	5 (4.2)
	Insulin X 1	1 (0.8)
	Insulin X 4	17 (14.2)
A1C, mean (SD)		7.7 (1.4)
A1C		
	< 7%	45 (37.5)
	> = 7%	75 (62.5)
Glucose (before meal), mean (SD)		145.3 (40.2)
Glucose (after meal), mean (SD)		173.2 (53.4)
Smoking		25 (20.8)

The participants HL and SE scores, as well as the scores in the DMSES subscales, are presented in Table 2. The mean SE score was 73.6 (SD = 9.3) and the mean HL score was 12.7 (SD = 2.3).

**Table 2:** Mean and median scores for HL, SE SD - Standard Deviation, IQR - Interquartile Range, SE - Self-Efficacy, HL - Health Literacy

	Mean (SD)	Median (IQR)
SE- Diet	29.0 (5.6)	29 (25.5 ─ 33)
SE- Medical therapy	20.4 (2.2)	21 (19 ─ 22)
SE- Medication and feet check	12.8 (1.2)	13 (12 ─ 14)
SE - Physical activity	11.4 (2.2)	12 (10 ─ 13)
SE total	73.6 (9.3)	74 (67.5 ─ 80.5)
HL	12.7 (2.3)	13 (11 ─ 15)

Based on the participants’ HL scores, we found that 54.2% of them had sufficient HL, 40% problematic, and 5.8% inadequate.

A1C was found significantly and negatively correlated with SE scores related to diet and physical activity and the SE total score, indicating higher SE in patients with lower A1C. Also, HL was found positively and significantly associated with greater SE (Table 3).

**Table 3:** Spearman’s correlation among A1C, HL, and SE scores Statistical significance was set at p < 0.05. A1C- Glycosylated hemoglobin, HL - Health Literacy, SE - Self-Efficacy, Rho - Spearman correlations coefficients

	A1C		HL	
	Rho	P	rho	P
SE - Diet	-0.22	0.016	0.42	< 0.001
SE - Medical therapy	-0.16	0.073	0.31	< 0.001
SE - Medication and feet check	-0.08	0.370	0.47	< 0.001
SE - Physical activity	-0.22	0.018	0.43	< 0.001
SE total	-0.26	0.005	0.50	< 0.001
HLS-EU-Q16	-0.09	0.315	-	-

Comparing the SE scores between patients with A1C < 7% and A1C > 7%, it was found that the SE-diet as well as the total SE score differed significantly between patients with < 7% A1C and those with ≥ 7% A1C, with patients with < 7% having significantly greater SE (Table 4).

**Table 4:** HL and SE scores according to A1C levels Statistical significance was set at p < 0.05. HL - Health Literacy, SE - Self-Efficacy, A1C- Glycosylated hemoglobin, SD - Standard Deviation, IQR - Interquartile Range

	A1C				P
	< 7%		≥ 7%		
	Mean (SD)	Median (IQR)	Mean (SD)	Median (IQR)	
SE - Diet	30.4 (5)	31 (28 ─ 33)	28.3 (5.8)	28 (24 ─ 32)	0.050
SE - Medical therapy	20.6 (2.1)	21 (19 ─ 22)	20.3 (2.2)	21 (19 ─ 22)	0.471
SE - Medication and feet check	12.9 (1.2)	13 (12 ─ 14)	12.7 (1.2)	13 (12 ─ 14)	0.364
SE - Physical activity	11.8 (2.1)	12 (11 ─ 13)	11.2 (2.3)	11 (10 ─ 13)	0.092
SE total	75.7 (8.6)	77 (71 ─ 82)	72.4 (9.5)	73 (67 ─ 80)	0.037
HL	12.9 (2.4)	14 (11 ─ 15)	12.5 (2.3)	13 (11 ─ 14)	0.318

When multiple regression was applied, it was found that HL was significantly associated with age, gender, and educational status. Women had significantly poorer HL compared to men as well as older patients. On the contrary, a higher educational level was significantly associated with greater HL (Table 5).

**Table 5:** Multiple linear regression results with HL as dependent variable Analysis was conducted after the logarithmic transformation of the dependent variable. +regression coefficient (Standard Error) ^1^scale from 1(None) to 4(University/MSc) ^2^scale from 1(< 400) to 4(> 1,500). Statistical significance was set at p < 0.05. HL - Health literacy, BMI - Body mass index

	β (SE)+	P
Age	-0.002 (0.001)	0.002
Gender (women vs men)	-0.046 (0.014)	0.001
Family status (Unmarried/ Divorced/ Widowed vs Married/ Living with partner)	0.003 (0.018)	0.859
Educational status^1^	0.030 (0.011)	0.005
Monthly income^2^	0.000 (0.009)	0.974
ΒΜΙ (Kg/m^2^)	0.000 (0.001)	0.896
Chronic disease (yes vs no)	-0.017 (0.027)	0.540

BMI and HL were significantly associated with all SE scores, after adjusting for all other characteristics. Thus, greater BMI was significantly associated with less SE. On the other hand, greater HL was significantly associated with greater SE (Table 6).

**Table 6:** Multiple linear regression results with SE scores as dependent variables Analysis was conducted after logarithmic transformation of the dependent variables+regression coefficient (Standard Error) ^1^scale from 1 (None) to 4 (University/MSc) ^2^scale from 1 (< 400) to 4 (> 1,500). Statistical significance was set at p < 0.05. SE - Self-Efficacy, BMI - Body mass index, HL - Health literacy.

	SE - Diet		SE - Medical therapy		SE - Medication and feet check		SE - Physical activity		SE total	
	β(SE)+	P	β(SE)+	P	β(SE)+	P	β(SE)+	P	β(SE)+	P
Age	0.000 (0.001)	0.591	0.000 (0.000)	0.454	-0.001 (0.000)	0.206	0.000 (0.001)	0.937	0.000 (0.001)	0.586
Gender (women vs men)	0.000 (0.018)	0.990	0.001 (0.010)	0.910	-0.01 (0.008)	0.213	0.010 (0.021)	0.622	0.000 (0.011)	0.998
Family status (Unmarried/ Divorced/ Widowed vs Married/ Living with partner)	0.015 (0.022)	0.499	0.010 (0.012)	0.390	0.002 (0.010)	0.810	-0.023 (0.026)	0.367	0.007 (0.013)	0.611
Educational status^1^	-0.023 (0.014)	0.086	0.005 (0.007)	0.467	-0.003 (0.006)	0.648	0.018 (0.016)	0.264	-0.004 (0.008)	0.609
Monthly income^2^	0.004 (0.011)	0.675	0.000 (0.006)	0.974	-0.004 (0.005)	0.453	-0.020 (0.012)	0.119	-0.001 (0.006)	0.852
ΒΜΙ (Kg/m^2^)	-0.003 (0.001)	0.029	-0.002 (0.001)	0.001	-0.002 (0.001)	0.010	-0.003 (0.001)	0.024	-0.003 (0.001)	0.001
Chronic disease (yes vs no)	-0.053 (0.034)	0.128	-0.010 (0.018)	0.589	-0.007 (0.016)	0.653	-0.042 (0.040)	0.291	-0.031 (0.020)	0.121
Complications of diabetes (yes vs no)	-0.005 (0.016)	0.772	-0.004 (0.008)	0.601	0.005 (0.007)	0.445	-0.029 (0.018)	0.118	-0.007 (0.009)	0.471
Smoking (yes vs no)	-0.020 (0.020)	0.323	0.003 (0.011)	0.801	0.010 (0.009)	0.283	0.004 (0.023)	0.861	-0.003 (0.012)	0.775
HL	0.018 (0.004)	< 0.001	0.006 (0.002)	0.004	0.008 (0.002)	< 0.001	0.014 (0.005)	0.004	0.011 (0.002)	< 0.001

To examine the mediating role of SE in the relationship between HL and A1C, a hierarchical linear regression analysis was conducted. HL was negatively associated with A1C at the 1st step of the analysis. After the total SE score was entered into the analysis (step 2), HL continued to be significantly associated with A1C, and the total SE score was found to be also significantly associated with A1C. Thus, SE partially mediates the relationship between HL and A1C, in a significant way based on the Sobel test (p = 0.046). Additionally, greater age was significantly associated with greater A1C. Women had significantly lower A1C compared to men and unmarried/ divorced/ widowed participants had significantly greater A1C compared to participants who were married or living with their partner.

Also, neither HL (OR = 0.93; 95% CI: 0.80-1.09; p = 0.352) nor total SE score (OR = 0.97; 95% CI: 0.93-1.01; p = 0.138) was significantly associated with having any complication of diabetes, thus no mediating effect of total SE score in the relationship of HL and having any complication of diabetes is present (Table 7).

**Table 7:** Multiple hierarchical linear regression results with A1C as dependent variable Analysis was conducted after the logarithmic transformation of the dependent variable. +regression coefficient (Standard Error) ^1^scale from 1(None) to 4(University/MSc) ^2^scale from 1 (< 400) to 4 (> 1,500). Statistical significance was set at p < 0.05. A1C- Glycosylated hemoglobin, BMI - Body mass index, HL - Health literacy, SE - Self-Efficacy.

Step		β(SE)+	P
1: F(8,111) = 7.49; p < 0.001; R^2 ^= 0.30	Age	-0.003 (0.001)	< 0.001
	Gender (women vs men)	-0.031 (0.013)	0.020
	Family status (Unmarried/ Divorced/ Widowed vs Married/ Living with partner)	0.049 (0.016)	0.003
	Educational status^1^	-0.009 (0.010)	0.357
	Monthly income^2^	-0.012 (0.008)	0.133
	ΒΜΙ (Kg/m^2^)	0.002 (0.001)	0.023
	Chronic disease (yes vs no)	-0.008 (0.024)	0.730
	HL	-0.009 (0.003)	0.003
2: F(9,110) = 7.20; p < 0.001; R^2 ^= 0.32, R^2^ change =.02	Age	-0.003 (0.001)	< 0.001
	Gender (women vs men)	-0.031 (0.013)	0.018
	Family status (Unmarried/ Divorced/ Widowed vs Married/ Living with partner)	0.050 (0.016)	0.002
	Educational status^1^	-0.010 (0.010)	0.309
	Monthly income^2^	-0.012 (0.008)	0.124
	ΒΜΙ (Kg/m^2^)	0.002 (0.001)	0.119
	Chronic disease (yes vs no)	-0.016 (0.025)	0.503
	HL	-0.007 (0.003)	0.039
	SE total	-0.002 (0.001)	0.030

## Discussion

According to the results of the study, HL is positively associated with SE, a finding that is confirmed by other studies in T2DM patients [34,35]. We also found SE to be associated with glycemic control a finding consistent with the literature [22,36]. According to Lee et al. [34] HL not only directly affects self-care activities, but also has an indirect effect on them, via self-efficacy. These findings may indicate that patients who can find, understand, and use health information have more confidence in themselves, develop skills, and adopt health behaviors that lead them to better disease management. 

In our study, the overall SE score differed significantly between patients who did or did not achieve the glycemic goal. Patients with A1C < 7% had better SE than those with A1C ≥ 7, a finding that confirms the relationship and is consistent with the study of Brown et al. [37]. We found HL to be associated with better A1C and a positive effect of HL on SE, findings consistent with the literature. Lee et al. [38] determined significant direct pathways from HL to SE, from SE to self-care behaviors, and from self-care behaviors to HbA1c levels. These findings may lead to the conclusion that a high level of HL influences SE by providing individuals with the knowledge, skills, and confidence needed to actively manage their T2DM. Also, we found that SE partially mediates the relationship between HL and A1C, while Osborn et al. [22] reported its indirect effect. The relationship between HL and SE as an interactive relationship is someway related to glycemic regulation. This relationship between HL and SE underscores the importance of promoting HL from health professionals to achieve diabetes therapeutic goals. 

Higher BMI was associated with lower SE. SE is a predictor of adherence behaviors such as diet and exercise, which is in agreement with our findings [39].

We also found that 54.2% of the participants had adequate HL, a percentage which is close to the average of the general population according to the HLS-EU study [31] (adequate/exceptional HL 52.5%), a study conducted in eight European countries in which also participated. The results from the Greek population sample are similar to ours (adequate/excellent HL 55.2%). According to a meta-analysis of 33 studies 67% of the patients with diabetes T2DM were found to have adequate HL [26]. The lower percentage of adequate HL in our study can be explained by the characteristics of the study population, as the participants' lower socioeconomic status compared to the general population.

A common finding of several researchers like ours who investigated the factors influencing HL is that it is related to age, educational level, and sometimes health behaviors such as dietary and physical activity, which in turn contribute to the achievement of A1C goals [40-41]. A study evaluating HL in an adult population in the USA showed that women had higher average HL than men [42]. Studies from Turkey [43] and Taiwan [44] conducted among women showed inadequate HL in 76.5% and 66.9% respectively. In the present study, women had statistically significantly lower HL than men. A systematic review of systematic reviews suggests that the differences in HL between the sexes need to be further investigated [45].

Gender differences in patients with type 2 diabetes also found in A1C values ​​​​with women having better A1C than men. According to a Canadian study [46], differences in A1C levels between the sexes are also present in other countries, with women in some having better values ​​and in some worse than men, while in others no difference was found. These differences may be due on the one hand to the levels of equality between the two sexes and on the other hand to the particular socio-economic and cultural characteristics of each population.

Socio-demographic characteristics such as age and marital status were associated in several studies with A1C rates. Older patients had better [47,48] A1C rates a finding that was not confirmed in our study. Marital status appears to influence health behaviors and outcomes including glycemic control. Despite the small number of patients living without a partner in our study, similarly to other studies, [49] we found that they have higher A1C values compared to those who were married or living with a partner.

Limitations

This study has certain drawbacks that limit its external validity. The small sample size (N = 120), the composition of the population (semi-urban, rural), and the fact that the sample comes from a diabetic clinic of one hospital in Greece does not allow us to make generalizations.

## Conclusions

The study emphasizes the significance of HL and SE in effectively managing T2DM. Implementing multi-tiered educational interventions for T2DM patients has the potential to enhance HL and SE, as well as encourage the practice of diabetes self-management. Additional investigation into the disparities in HL levels between males and females has the potential to inform the development of health strategies aimed at mitigating the discrepancy. Moreover, the prospect of reaching an agreement across several disciplines regarding the incorporation of SE measurement methods and HL in patients with diabetes is very intriguing. Effectively integrating educational strategies to enhance both SE and HL is a significant challenge, but also crucial to effectively promote diabetes self-management.
